# High-flux ultrafast extreme-ultraviolet photoemission spectroscopy at 18.4 MHz pulse repetition rate

**DOI:** 10.1038/s41467-019-08367-y

**Published:** 2019-01-28

**Authors:** T. Saule, S. Heinrich, J. Schötz, N. Lilienfein, M. Högner, O. deVries, M. Plötner, J. Weitenberg, D. Esser, J. Schulte, P. Russbueldt, J. Limpert, M. F. Kling, U. Kleineberg, I. Pupeza

**Affiliations:** 1 Max-Planck-Institut für Quantenoptik (MPQ), Hans-Kopfermann-Str. 1, 85748 Garching, Germany; 2 Ludwig-Maximilians-Universität München (LMU), Am Coulombwall 1, 85748 Garching, Germany; 3 Fraunhofer-Institut für Angewandte Optik und Feinmechanik (IOF), Albert-Einstein-Str. 7, 07745 Jena, Germany; 4 Fraunhofer-Institut für Lasertechnik (ILT), Steinbachstr. 15, 52074 Aachen, Germany; 5 Friedrich-Schiller-Universität Jena, Institut für Angewandte Physik (IAP), Albert-Einstein-Str. 15, 07745 Jena, Germany; 6 Helmholtz-Institut Jena, Fröbelstieg 3, 07743 Jena, Germany; 7 Active Fiber Systems GmbH (AFS), Wildenbruchstr. 15, 07745 Jena, Germany

## Abstract

Laser-dressed photoelectron spectroscopy, employing extreme-ultraviolet attosecond pulses obtained by femtosecond-laser-driven high-order harmonic generation, grants access to atomic-scale electron dynamics. Limited by space charge effects determining the admissible number of photoelectrons ejected during each laser pulse, multidimensional (i.e. spatially or angle-resolved) attosecond photoelectron spectroscopy of solids and nanostructures requires high-photon-energy, broadband high harmonic sources operating at high repetition rates. Here, we present a high-conversion-efficiency, 18.4-MHz-repetition-rate cavity-enhanced high harmonic source emitting 5 × 10^5^ photons per pulse in the 25-to-60-eV range, releasing 1 × 10^10^ photoelectrons per second from a 10-µm-diameter spot on tungsten, at space charge distortions of only a few tens of meV. Broadband, time-of-flight photoelectron detection with nearly 100% temporal duty cycle evidences a count rate improvement between two and three orders of magnitude over state-of-the-art attosecond photoelectron spectroscopy experiments under identical space charge conditions. The measurement time reduction and the photon energy scalability render this technology viable for next-generation, high-repetition-rate, multidimensional attosecond metrology.

## Abstract (web summary)

Space charge effects can distort the results of photoelectron spectroscopic measurements, and usually limit the allowable photon flux in an experiment. Here, the authors present an 18.4 MHz repetition rate high harmonic source in the 25–60 eV range, with a large count rate improvement over state-of-the-art attosecond setups under identical space charge conditions.

## Introduction

At the beginning of this century, a series of seminal technological developments in the field of ultrafast lasers (reviewed, e.g., in ref. ^1^), led to the first photoelectron spectroscopy^2^ (PES) experiments performed with extreme-ultraviolet (XUV) radiation obtained by high-order-harmonic generation (HHG) driven by intense, visible/near-infrared (VIS/NIR) pulses in gases, in the presence of the (delayed) driving field^3,4^. In these experiments, the sub-optical-cycle temporal structure of HHG radiation, and its synchrony with the driving field providing a temporal reference, enabled laser-dressed XUV-PES with sub-femtosecond temporal resolution, marking the birth of attosecond metrology^1^. The two major PES-based tools employed in this emerging field, attosecond streaking^1,5–7^, using isolated attosecond pulses, and RABBITT (reconstruction of attosecond harmonic beating by interference of two-photon transitions)-based techniques^8^, using attosecond pulse trains^4,9–15^, rely on analysing the evolution of photoelectron spectra with varying delay between the two optical fields. Both approaches have been widely applied for measurements of attosecond photoemission delays from gases, providing insights into electron dynamics deep inside the atom (see e.g., refs. ^1,7,13,14,16^ and references therein). During the past decade, considerable efforts have addressed the extension of these techniques to solids^6,9–12^ and nanostructures^17,18^. For instance, very recently the combination of angle-resolved PES (ARPES) and RABBITT has enabled first band-specific measurements of correlated electron dynamics on metal surfaces^9,10^. Another example is the on-going effort to combine attosecond streaking with photoemission electron microscopy (PEEM) for the spatiotemporal study of complex plasmonic fields propagating on nanostructured metal surfaces^17–21^. Fundamental studies of correlated electron dynamics in these dense atomic systems are necessary for the development of novel materials and high technologies.

**Fig. 1:**
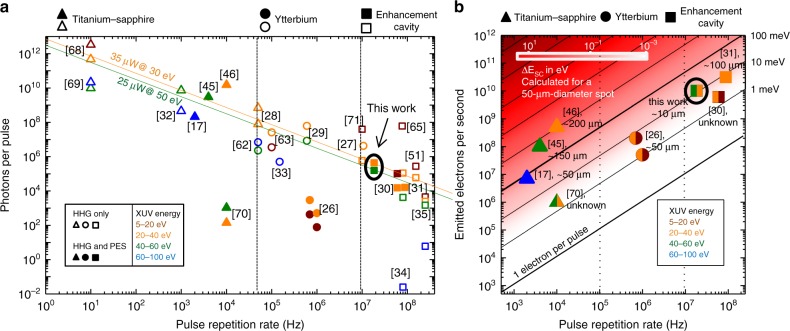
Flux of state-of-the-art high-harmonic generation sources and PES experiments. **a** Generated XUV photons per pulse versus pulse repetition rate for different energy ranges (colour-coded, see legend) in state-of-the-art HHG sources (representative selection). The shape of the symbols (top of the figure) indicates the underlying femtosecond laser technology. Full and empty symbols indicate HHG sources which have and which have not been used for PES, respectively. The diagonal lines represent lines of constant XUV flux at a given energy: 30 eV (orange) and 50 eV (green). Data from refs. ^17,26–35,45,46,51,62,63,65,68–71^ **b** Photoelectron flux and estimated space-charge-induced kinetic energy distortion ΔE_SC_ (i.e., spectral broadening and energy shift of the same order, see Methods) in state-of-the-art ultrafast PES experiments versus pulse repetition rate. The colours of the symbols indicate different photon energy ranges (see legend). For each setup, the spot diameter employed in the experiment is given, where available. For comparison, the energy distortion (heat map) is calculated relating the number of released photoelectrons to a 50-µm-diameter spot (typical size). For photon energies higher than 40 eV (typical for attosecond-PES), a space-charge-limited spectral resolution of 0.1 eV corresponds to a number of ~10^4^ photoelectrons released from the sample for each XUV pulse, resulting in an admissible number of ~1.5 × 10^5^ impinging photons (see Methods). Data from refs. ^17,26,30,31,45,46,70^ Note, that the photoelectron flux in refs. ^26,30,31,46,70^ is generated by single-harmonic XUV excitation

A severe shortcoming of laser systems employed in state-of-the-art attosecond-PES experiments, however, arises from their relatively low pulse repetition rate *f*_rep_, usually well below 1 MHz, in the context of space charge^22,23^ (SC). Coulomb interaction of multiple photoelectrons released from the sample during a single pulse affects their velocities and trajectories towards the detector, leading to distortions of the observables, i.e., kinetic energy (and momentum or position)^17,23^. In fact, in the vast majority of setups for (multidimensional—i.e., energy, and angularly or spatially resolved) attosecond-PES on solids, space charge effects demand a significant attenuation of the HHG output^9,17^. At a space-charge-limited photoelectron flux and at typical *f*_rep_ values in the kHz range, the acquisition of (multidimensional) photoelectron spectra with sufficient signal-to-noise ratio necessitates impractically (or even prohibitively) long acquisition times of several hours^10^ to several tens of hours^17^. Over such periods, laser instabilities and sample contamination^24^ constitute severe technological challenges. This shortcoming can potentially be circumvented by HHG sources with repetition rates in the multi-MHz range. Fig. 1a shows an overview of state-of-the-art HHG sources illustrating that to date, however, the combination of high photon energies, high photon flux and high repetition rate has remained an unmet challenge. Although large-scale technologies such as FELs also offer high flux at high photon energies and repetition rates^25^ to date, they do not enable measurements with attosecond time resolution.

Here, we close this gap with a high-conversion-efficiency cavity-enhanced HHG source. Driving HHG at *f*_rep_ = 18.4 MHz with sub-40-fs, 1-µm pulses in argon results in XUV pulse trains with photon energies between 25 and 60 eV and, in particular, a record flux at >40 eV for high *f*_rep_ (Fig. 1a). In experiments demonstrating the potential of this source for time-resolved PES, a 10-µm-diameter spot on a tungsten surface was illuminated, generating between two and three orders of magnitude more photoelectrons than affordable in previous state-of-the-art, high-photon-energy attosecond-PES experiments under the same space-charge-imposed restrictions (Fig. 1b). Time-of-flight (ToF) detection allows for the simultaneous collection of photoelectrons from a large solid angle and in a broad range of kinetic energies, with a temporal detection duty cycle close to 100% (i.e., the time of flight dispersion corresponds to the pulse repetition period). Unlike other detection schemes (e.g., hemispherical or retarding-field analyzers), ToF spectrometers allow for the simultaneous detection of electron emission momentum (or spatial distribution) and kinetic energy without the need to scan over one of these parameters at the expense of a reduced flux. Furthermore, attosecond temporal resolution is inferred from the sideband modulation of laser-assisted photoemission^4^.

## Results

### Cavity-enhanced HHG

Modern MHz-repetition-rate ytterbium-based lasers (see refs. ^26,27^ and references therein) currently hold average power records for single-pass HHG with photon energies up to around 40 eV^27–29^, obtained from krypton and xenon (Fig. 1a), and have been successfully employed for narrowband PES measurements at these photon energies, both in single-pass^26^ and in cavity-enhanced HHG configuration^30,31^ (Fig. 1b).

However, with these technologies significantly higher photon energies (for which gases with a higher ionisation potential like argon or neon are typically employed) were so far only achieved at the cost of the repetition rate in single-pass HHG^17,32,33^ or of the photon flux in cavity-enhanced HHG^34,35^, see Fig. 1a. Here, we demonstrate that a combination of state-of-the-art fibre laser technology, nonlinear pulse compression and cavity-enhanced HHG can overcome these limitations.

**Fig. 2:**
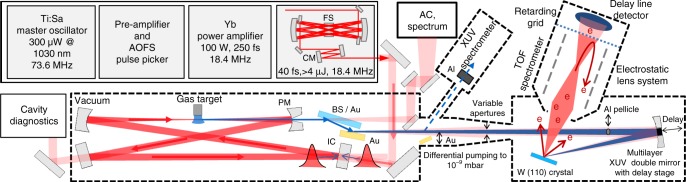
Experimental setup. The MOPA generates a train of 18.4-MHz-repetition-rate, 250-fs pulses centred at 1030 nm. The output pulses are nonlinearly compressed to < 40 fs via spectral broadening upon multiple-pass self-phase modulation in fused silica (FS) followed by a chirped-mirror (CM) compressor, and coherently enhanced in a femtosecond enhancement cavity. HHG is driven in a 25 × 32-µm^2^-radius focus between two curved mirrors, one of which is pierced for on-axis XUV output coupling. IC: input coupler, PM: pierced mirror. Intracavity pulse diagnostics: autocorrelator (AC), optical spectrum analyser and power meter. The output-coupled XUV can either be sent to an XUV spectrometer or to the PES experiment. A 96%-NIR-transmission beam splitter (BS) can be used to separate the NIR radiation from the co-propagating XUV beam. Alternatively, the BS can be exchanged with an Au mirror, resulting in 10^11^ W/cm^2^ NIR intensity on the sample for laser-dressed PES. In the ultra-high-vacuum PES chamber (<10^−9^ mbar) the XUV beam is focused onto the tungsten (110) crystal with a 250-mm radius-of-curvature multilayer (scandium-silicon) double mirror. The inner segment of the mirror can be delayed with a piezoelectric-ceramic stage. A moveable on-axis aluminium filter can optionally block the NIR otherwise impinging on the inner mirror segment. Fine-tuning the NIR power is achieved with a variable aperture. The electrons are detected by a time-of-flight spectrometer equipped with a retarding grid. Two operation modes are available, the drift mode without any manipulation of the electron trajectory and the low-angular-dispersion mode with capture in a larger solid angle (see Methods)

**Fig. 3:**
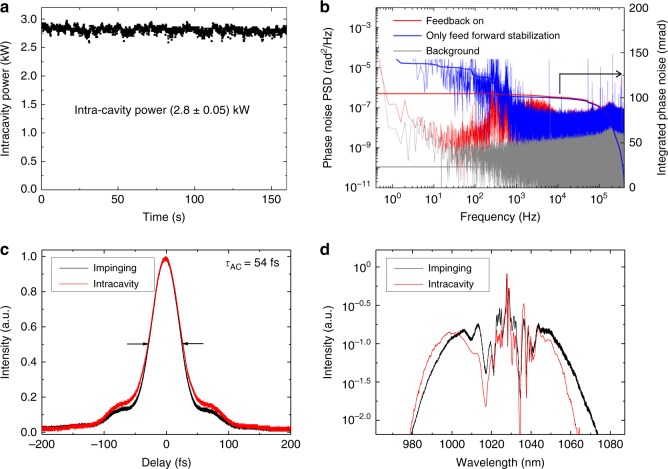
Laser system and intra-cavity specifications. **a** Measurement of the nonlinear intracavity power over a duration of 160 s, under experimental conditions for optimised HHG generation. The root-mean-square intensity fluctuations amounts to 1.8%. **b** Phase noise measurement of the MOPA output. Feed-forward phase stabilisation of the pulse train before the CPA^36^ results in an phase noise <150 mrad integrated in the band of 0.4 Hz–400 kHz (blue). Employing a feed-back loop for phase noise compensation after the CPA and nonlinear compression reduces the integrated phase noise to ~100 mrad (red). However, this additional stabilisation was not used in the PES experiments, as it did not improve the PES statistics. **c** Autocorrelation traces of the impinging pulses (black) and the pulses circulating in the EC with an argon target (red). In the case of the argon target, a slight temporal broadening is observed, which can be attributed to the nonlinear interaction of the circulating pulse with the gas target. The measured full-width-at-half-maximum of the autocorrelation trace (τ_AC_ = 54 fs) indicates a sub-40-fs duration for both pulses. **d** Spectra of the fundamental light impinging the EC (black) and the circulating light with an argon target (red). The blueshift typical to intra-cavity HHG^38^ can be observed

The experimental setup is depicted in Fig. 2. The HHG source (see also Methods) consists of a master-oscillator-power-amplifier (MOPA) femtosecond frontend followed by a multi-pass bulk-compression stage and a femtosecond enhancement cavity (EC) housing the HHG gas target. The initial pulses were delivered by a titanium-sapphire oscillator with a repetition rate of 73.6 MHz. By means of a pulse picker after the oscillator, the repetition rate of the MOPA output^36^ was reduced to a quarter to permit sufficient energy dispersion in ToF-PES. The output of the MOPA was an 18.4-MHz train of 5.4-µJ, 250-fs pulses, spectrally centred at 1030 nm. Multi-pass nonlinear spectral broadening in bulk and subsequent chirp removal compensation with dispersive mirrors allowed for the temporal compression of these pulses down to sub-40-fs duration^37^ at an energy level up to one order of magnitude higher than in previous cavity-enhanced HHG setups with comparable pulse durations (see refs. ^34,35^ and references therein). After chirped-pulse amplification the pulses were coherently overlapped in a passive, 16.3-m roundtrip bowtie EC with a finesse of 140. At a power enhancement of 35, inside the EC, a 0.15-mJ, sub-40-fs circulating pulse was stably maintained over several minutes (Fig. 3a). The key technological aspect to achieving this high intensity and phase stability was using a feed-forward phase-stabilised Ti:Sa oscillator to seed the Yb-fibre amplifier^37^ (see Fig. 3b) and actively controlling the repetition frequency of the seed oscillator and the EC length with a fast and a slow loop, respectively. High harmonics of the circulating NIR radiation were generated in an argon gas target at the focus, and coupled out through a 340-µm-diameter on-axis opening in the resonator mirror following the focus^34,35^. Fig. 3c, d show the autocorrelation and the spectra of the pulses impinging and circulating in the EC, respectively.

**Fig. 4:**
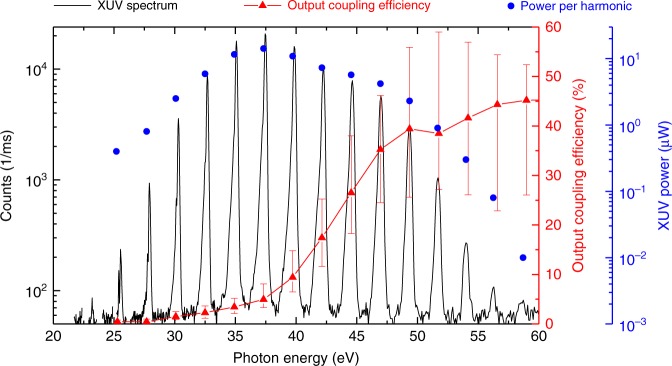
Output coupled XUV radiation. The spectrum (black) was recorded with an XUV grating spectrometer. After spectral filtering with a 300-nm aluminium filter, the total XUV power was measured with a calibrated photodiode (AXUV100 Al .3, Opto Diode Corp.). The given power in each harmonic is the output coupled power right behind the pierced mirror which was corrected for filters and mirrors between that point and the spectrometer, and assuming a 100% diffraction and detection efficiency of the XUV spectrometer. The output coupling efficiency (red) was calculated with a 3+1 D HHG model for a nozzle position one Rayleigh range before the focus (see Methods)

Argon gas was delivered through an end-fire quartz nozzle with a 200-µm-diameter opening. The gas target position and the backing pressure were optimised for highest XUV yield around 40 eV after output coupling. An output coupled XUV flux of 1 × 10^13^ photons/second was measured, with energies ranging from 25 to 60 eV (Fig. 4). By combining state-of-the-art fibre laser technology with a novel nonlinear pulse compression scheme, a pulse energy of 4.5 µJ was available as a seed to the enhancement cavity, leading to 0.15-mJ intracavity pulses. Importantly, the moderate cavity finesse alleviates ionisation-related intensity limitations (clamping) that arise from the blueshift caused by self-phase-modulation in the gas target^38–40^. Together with relatively weak cumulative ionisation effects in the gas target (each atom traverses the beam diameter within 4 laser shots), this enabled an intracavity conversion efficiency around 10 times higher than in^34,35^ and, for the first time, approaching that obtained with single-pass HHG in argon (see Methods). This allowed for a highly efficient use of the EC concept, in particular leading to – to our knowledge – the highest usable XUV flux for photon energies beyond 40 eV at *f*_rep_ > 1 MHz reported so far (see Fig. 1).

### Ultrafast photoelectron spectroscopy

**Fig. 5:**
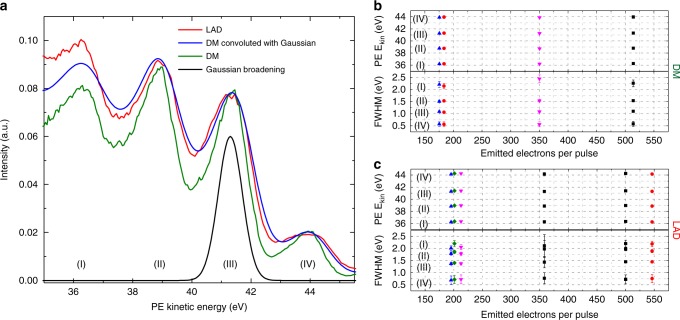
Photoelectron spectral features in different detection modes and at different photoelectron flux. **a** Photoelectron spectra taken with two different detection modes: drift mode (DM) and large angular dispersion (LAD) mode (see Methods). In the DM the photoelectrons propagate with their initial energy and direction from the sample to the detector (acceptance angle of 0.0016 sr) without any additional acceleration or deflection, thus providing pristine detector energy resolution of 30 meV. In the LAD mode, the capture angle is increased to 0.047 sr by a complex electron lens system. The energy resolution of the LAD mode is obtained by convolution of the DM spectrum (having a well-known detector resolution of 30 meV) with a 1-eV full-width-at-half-maximum Gaussian, which reproduces the LAD spectrum well. **b**, **c** Show the evaluation of the peak positions and widths of the characteristic spectral fringes, for different XUV intensities on the sample, for the DM and the LAD modes, respectively. Attenuating the XUV flux was achieved by clipping the beam with a hard aperture. The error bars illustrate the error of the peak fit. Neither the DM nor the LAD mode show space charge effects

For PES experiments, the XUV beam and, optionally, the NIR beam, were directed through a differentially pumped beamline and focused to a 10-µm-diameter spot onto the surface of a single-crystalline (110) tungsten target with a multilayer XUV mirror (Fig. 2). Photoelectrons were collected with a ToF spectrometer (equipped with a retarding grid) with two settings—drift mode (DM) and large-angular-dispersion mode (LAD)—differing in their capture angle and energy resolution (see Fig. 5a and Methods).

A first PES experiment was aimed at measuring the number of photoelectrons released from the sample and scrutinising the space-charge-induced spectral distortions. To this end, the XUV beam was separated from the 6-W average power NIR beam passing through the opening in the output-coupling mirror with a beam splitter. Thus, all of the detected photoelectrons stem from single-photon XUV photoemission. Although the use of XUV transmission optics was avoided, the reflective optics attenuated the XUV photon flux by 97%, resulting into 3 ×10^11^ photons/second at the sample, releasing an estimate of around 1 × 10^10^ photoelectrons/second. This value is in good agreement with that calculated from the ToF count rate, and results in an estimated space charge distortion of ΔE_SC_≈50 meV (see Methods). In accordance with the instrument resolution of 0.13–0.36 eV (limited by the spectral width of the harmonics, see Methods), no distortions of the spectra were discernible for both acquisition modes (see Fig. 5b, c).

**Fig. 6:**
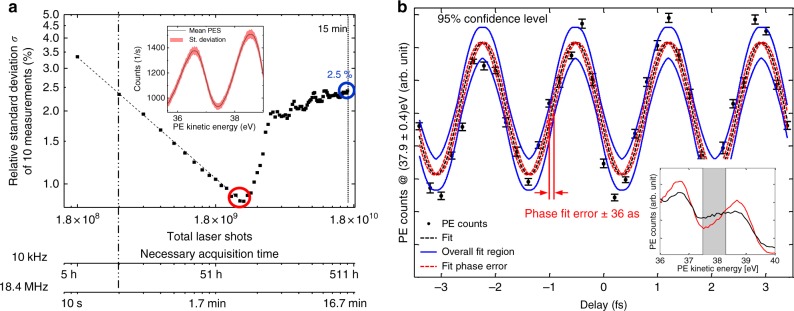
Photoelectron statistics and time-dependent sideband modulation. **a** Measurement over ~15 min (1.8 × 10^10^ laser shots). The plot shows the evolution of the relative standard deviation σ of the counts (averaged from 35 to 44 eV kinetic energy). The dashed line is a 1/$\sqrt T$-fit of the first 160 s. The excellent agreement confirms statistical behaviour up to 160 s with a minimum relative standard deviation of 0.9% for this measurement time (red circle). For *T* > 160 s, slow drifts emerge. The inset shows the mean (black) and relative standard deviation (red) of a PES measurement with 160 s. The bottom axes illustrate the time a 10 kHz system would need for the same amount of laser shots. **b** Intensity of the sideband at 37.9 eV kinetic energy as a function of the NIR-XUV delay taken within 105 s in total. The phase fit error (red dashed line) corresponds to 36 as and is comparable to the timing jitter of the interferometer (see Methods). Blue lines show the maximum and minimum error boundaries of all fit parameters. The inset shows photoelectron spectra at two different delays with (black) and without (red) sideband and the spectral region over which the sideband intensity was integrated. The error bars have been calculated by dividing the dataset into 10 subsets, calculating the relative standard deviation of the 10 resulting data points at each delay step and dividing it by $\sqrt {10}$ to account for the better statistics of the entire dataset

To evaluate the statistics of the PES measurements we calculated the relative standard deviation σ of several measurements, for varying integration time *T* of each measurement. The results are shown in Fig. 6a. A statistical behaviour (decrease of σ with $\sqrt T$) was observed for *T*< 160 s, resulting in σ < 1%. Long-term drifts led to an increase of σ to ~2.5% after *T* = 15 min.

**Fig. 7:**
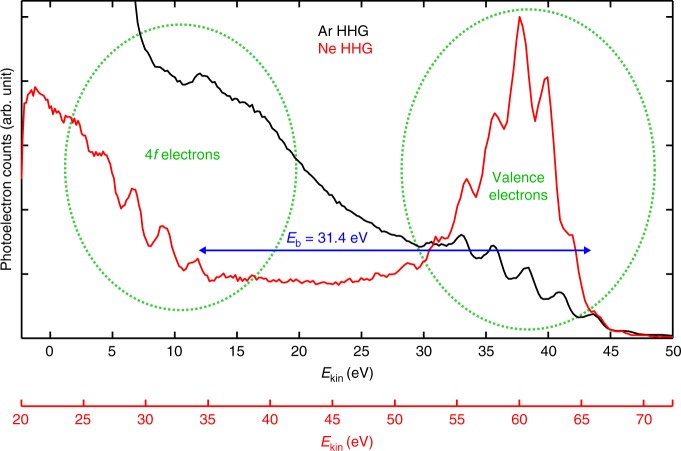
Photoelectron spectra from the high-harmonic generation in argon and neon. For spectral filtering the neon (argon) harmonics, an XUV multilayer mirror centred at 65 eV (49 eV) was used. In both cases, photoelectron peaks stemming from the tungsten valence band and the 4f core state are clearly visible and separated by the 4f 7/2 binding energy of 31.4 eV^42^. To account for the different excitation energies, the photoelectron spectra are shifted by 22.3 eV with respect to each other (note different x-axes). Results in argon are shown in black; neon in red

To demonstrate the suitability of the HHG source for attosecond-resolution PES, we performed XUV-pump-NIR-probe measurements. The XUV-NIR beam splitter was replaced by a gold mirror, enabling NIR intensities in the 10^11^-W/cm² range at the target. For introducing a mutual delay, the XUV and the NIR beams leaving the EC were spatially separated by a coaxial optic featuring a small 300-nm-thick aluminium filter covering the centre of the beam, held in position by thin metal wires. The outer part is transparent for the NIR pulses, whereas the central part is only transparent for the XUV. A variable temporal delay was introduced by a two-segment spherical mirror (Fig. 2). The fixed outer gold-coated ring reflects the NIR radiation and a movable, 5-mm-diameter multilayer-coated inner segment reflects the time-delayed XUV pulse trains with a peak reflectivity of 14% at 44 eV and in a bandwidth of 10 eV. This common-path geometry renders the XUV-NIR interferometer inherently stable. Fig. 6b shows the intensity of the sideband peak at 37.9 eV as a function of the pump-probe delay. Its delay-dependent oscillation at twice the fundamental laser frequency is caused by the interference of two different two-photon-transitions^4^. This sinusoidal curve (Fig. 6b) contains information on the relative time delay between the NIR and XUV fields, on the spectral phase of the adjacent harmonics, as well as on the delay of the photoemission process itself and, thus, on the underlying electron dynamics in the sample. Using the RABBITT-technique^4,8^, this information can be extracted from the phases of these sideband modulations^10,41^. In order to determine an upper bound of the time resolution of our instrument, a sine curve with three free parameters (amplitude offset and phase) at a fixed wavelength of 1030 nm was fitted to the data. The resulting fit error of the phase indicates a timing precision of the interferometer of 36 as, which is comparable to state-of-the-art attosecond-PES experiments^10,11^. Notably, the common-path XUV-NIR interferometer does not necessitate active stabilisation^10,11^ of the delay line. In the photoelectron spectra at a kinetic energy of 12 eV, a distinct peak that we attribute to the tungsten 4f doublet state is visible (Fig. 7). Its energy separation from the highest valence band peak corresponds well to the literature value of the binding energy of the tungsten 4f 7/2 state of 31.4 eV^42^. Since it is difficult to discern peaks below 12 eV due to the background of secondary electrons, we generated significantly higher harmonics in neon and used a multilayer mirror centred at 65 eV to spectrally filter them without changing anything within the cavity but the gas, its pressure and the position of the gas nozzle. As it can be seen in Fig. 7, the higher photon energies of neon HHG allow to clearly observe several photoelectron peaks originating from the 4f-state. Due to spin-orbit coupling, the 4f-state is split into two peaks separated by 2.2 eV which differs by only 0.2 eV from the separation of 2.4 eV between two high harmonics. Consequently, a photoelectron peak from the deeper bound 4f 5/2 state excited by photons of a specific high harmonic would be separated by only 0.2 eV from the photoelectron peak resulting from the 4f 7/2 state and photons from the next lower harmonic. Although this can hardly be distinguished by eye in this particular measurement, techniques to separate such features reliably in time-resolved measurements (e.g., in the Fourier space) have recently been developed and applied successfully^43,44^. The different appearance of the valence band electrons of neon and argon high harmonics can be explained by the better separation from the secondary electron background in combination with the narrower bandwidth of the XUV mirror.

## Discussion

We have presented a HHG source affording a combination of high repetition rate (*f*_rep_ = 18.4 MHz), high photon energies (up to 60 eV) and high photon flux (5 × 10^5^ photons per pulse). Proof-of-principle PES measurements on tungsten evidence the advantages of this parameter regime for PES-based attosecond metrology, over state-of-the-art HHG sources employed in this field. In particular, we demonstrated an improvement of the photoelectron flux by more than two orders of magnitude in space-charge-limited attosecond-PES. Considering the relatively strong attenuation of the XUV flux between the HHG source and the sample (almost two orders of magnitude), in principle an even higher photoelectron flux could be achieved with this HHG source. In addition, PES statistics attest the stability of this source for a comparatively very large number of laser shots. For measurement times of 160 s and 15 min, photoelectron spectra with standard deviations of less than 1% and ~2.5%, respectively, were taken. At our repetition rate, these two durations correspond to 2.9 × 10^9^ and 1.7 × 10^10^ shots, respectively. For comparison, at a repetition rate of 10 kHz, typical for state-of-the-art attosecond-PES experiments^9–11,13,14,17,45,46^, this would correspond to 3.4 and 19 days of continuous measurement, respectively. A particularly important aspect of the decrease in measurement time is the fact that this large number of shots can be recorded over a duration shorter than the typical sample contamination time under ultra-high vacuum conditions. Typically, at a pressure of 10^–10^ mbar, a monolayer of residual gas particles builds up in around 2 h^24^. This requires periodic cleaning, which considerably increases the overall measurement time and experiment complexity.

Furthermore, the measurement time reduction is expected to lead to a corresponding improvement of the temporal resolution in attosecond-PES experiments. In particular, the measurement of the sinusoidal oscillation of the laser-assisted XUV-photoemission sideband shown in Fig. 6b was done within 105 s which lies well within the time that the system behaves statistically (Fig. 5a), implying that multidimensional PES measurements can be repeated multiple times in the absence of drifts. The high flux and repetition rate of our system could even enable delay scans within less than a millisecond, which would decrease the impairment of the time resolution by acoustic vibrations. As was recently demonstrated^7^, averaging the results of a large number of measurements can enable the determination of photoionisation delays with absolute errors in the sub-attosecond range.

The current configuration of our apparatus is particularly suited for RABBITT-based attosecond metrology. For measurements of photoionisation delays, these techniques exhibit a few distinct advantages. For instance, they combine high temporal resolution, given by the individual attosecond bursts in the XUV pulse train, with high spectral resolution afforded by the discrete nature of the harmonics, and they offer high signal-to-noise ratios due to the evaluation of sideband modulations rather than shifts of broad photoelectron spectra^9–11,13,14^. In particular in combination with ARPES, very recently broadband RABBITT measurements have led to a number of insights into band-specific multi-electron attosecond correlation dynamics in solids^9–11^. The instrument presented here offers the prospect of a reduction of the measurement time in such investigations, promising the application of these attosecond tools to a wide range of material systems in the near future.

In contrast to previous multi-MHz HHG sources, our apparatus generates photons with energies of several tens of eV at a significantly higher flux. In addition, due to geometrical XUV output coupling, the architecture is photon-energy scalable to 100 eV or more^34,35^. Besides the general advantages of rendering PES less sensitive to external fields and of affording a better temporal resolution and a better energy separation of primary from secondary electrons, high photon energies are crucial for accessing deeply bound states of the system under study. This enables the comparison of photoemission delays from different initial states for self-referencing^6,7^ and is desirable for experiments on X-ray magnetic circular dichroism, e.g., at the M-edge of nickel or cobalt^47^. We demonstrated that the high photon energies of our setup enable addressing such a deeply bound state in tungsten, as it can be seen in Fig. 7. This may allow one to compare photoemission delays between the valence band and a deeper bound state in a solid material utilizing the RABBITT-technique.

The femtosecond-laser technologies presented here can be extended both to longer and to shorter^48,49^ pulses. Driving HHG with longer intracavity pulses is a way towards higher spectral resolution for RABBITT-based measurements. Shorter pulses, together with cavity-enhanced gating methods offer the prospect of the generation of temporally isolated attosecond pulses at multi-MHz repetition rates^50^. Such pulses could be used, for example, in multidimensional attosecond-streaking-based measurements, such as attosecond photoelectron emission microscopy of nanoplasmonic fields^17–19,21^. Beyond attosecond metrology the high-power multi-MHz-repetition-rate HHG source presented here could benefit applications such as XUV frequency comb spectroscopy^51^ for precision-measurements of electronic energy levels of atoms^52^, or for referencing to nuclear transitions^53^ for future nuclear clocks.

## Methods

### Estimation of space charge effects

The effect of space charge on the photoelectron spectra is estimated by using the Long-Itchkawitz-Kabler (LIK) model^54^. The model is based on the assumption of a radially expanding spherical, initially monoenergetic charge distribution and yields an energy broadening ΔE_SC_ of the spectrum given by ΔE_SC_[eV] = 3 × 10^−3^ N_e_/(0.5d_focus_[µm])^22,54^, where N_e_ denotes the number of emitted electrons per pulse and d_focus_ is the diameter of the emission spot. The validity of the model has been confirmed by classical many-body calculations. It has been shown to also be applicable to photoelectrons uniformly distributed in energy by changing the factor on the right-hand side of the above equation to 0.5 × 10^−3^(±0.12 × 10^−3^) for electrons on the high-energy side of the spectrum^22^. Furthermore, the simulations showed that the broadening is usually accompanied by an energy shift of roughly the same magnitude. We used this modified relation to estimate the space-charge-caused broadening. To relate the number of XUV photons to the number of emitted electrons, and to calculate the expected space charge effect for the various experiments shown in Fig. 1b (for a fixed spot size), the total photoemission probability including scattered and secondary electrons is needed. In the case of our experiment, we determined a photoemission probability of 3.3% from the measurement of the number of emitted electrons per pulse and taking into account the attenuation of ~97% of the output coupled XUV radiation along the beam path to the tungsten surface (the attenuations due to one Nb_2_O_5_ beam splitter, one gold mirror and a scandium-silicon XUV multilayer mirror amount to 50%, 40 and 90%, respectively). This value is in satisfactory agreement with the quantum efficiency of tungsten photocathodes experimentally determined previously to decrease from roughly 10% at 25 eV to about 6% at 50 eV^55^. The discrepancy can be attributed to the uncertainty of the attenuation estimation (including geometrical losses at the 5-mm-diameter XUV mirror, angle uncertainties, surface roughness etc.). For typical PES experiments, an energy resolution on the order of 100 meV is sufficient to distinguish different initial states in the electronic bandstructure of a solid^10^, which justifies the choice of the admissible space-charge-induced energy broadening in the discussion. Furthermore, we assumed a typical focal spot diameter of 50 µm for the comparison, while in our experiment, to scrutinise the presence of space charge effects, we utilized a smaller spot size of 10 µm in order to increase the sensitivity to these effects. In attosecond-PES, in order to have homogeneous conditions for all emitted electrons, the focal spot of the XUV focus is restricted to be smaller than the spot size of the pump beam. The latter, in turn, is limited by the requirement to provide high enough intensities to generate detectable signals. In case of RABBITT -experiments, NIR- intensities of 10^11^ W/cm² are well suited to generate a high flux of two-photon transitions while still preventing a significant portion of higher-order transitions. More generally in the case of non-homogenous samples (e.g. nanostructures for PEEM-experiments) such spot sizes are crucial.

In addition, the admissible electron flux might be limited by space-charge-induced distortions within the detector, e.g., if additional electron optics with intermediate foci are used. For instance, for attosecond photoemission electron microscopy it has been estimated, that the spatial resolution can be severely affected already at an electron flux of about 0.01 e/µm^2^^17^. This corresponds to only 20 electrons per pulse within a 50-µm-diameter emission spot, which is a factor of 20 below our electron flux, thus imposing even stronger limitations than in the example considered in Fig. 1b.

### High-flux, high-photon-energy 18.4-MHz HHG source

The seed for the MOPA system is a 300-µW, 73.6-MHz train of pulses spectrally centred at 1030 nm, split off from the output of a titanium-sapphire oscillator. Utilising a synchronous acousto-optic-frequency-shifter-based pulse picker^56^, a feed-forward carrier-envelope-offset (CEO) frequency stabilisation^57^ and three successive chirped-pulse Yb-doped fibre amplification stages (with temporal stretching to 200 ps), after the reflection-grating compressor an 18.4-MHz train of 5.4-µJ, 250-fs pulses is obtained. Previous studies have shown that the amplification and the pulse picking processes preserve the phase and intensity stability of the oscillator nearly perfectly^36,56^. The MOPA output is spectrally broadened by self-phase modulation in a multi-pass-cell with 49 passes through a 12-mm thick fused-silica medium and temporally compressed by dispersive mirrors to sub-40 fs duration. The efficiency of the nonlinear compression unit is 88%^37^. To minimise fluctuations of the spatial overlap between the compressed pulses and the EC at the input coupler, an active beam pointing stabilisation was installed, and the majority of the setup was placed in a housing to reduce air fluctuations. The bowtie enhancement cavity consisted of 8 mirrors, 7 of which are highly reflective quarter-wave stacks and one is a 97%-reflectivity input coupler. Taking into account the input power (80 W), the circulating average power (2.8 kW) and the overlap of 60%, we determine the finesse of the EC to be 140 and the losses at the pierced mirror to be 1.5%. This value is slightly higher than the expected losses of 1% our 4.2 × 3.1-mm-radius mode experiences at the 340-µm aperture in the XUV output coupler. This might be due to slight deformations of the pierced mirror around the whole. The output coupler was pierced by inverse laser drilling^58^. During the drilling, the substrate surface was protected by an optically bonded cover, leading to an excellent edge quality^59^. The focusing mirrors with a radius of curvature of 600 mm were chosen according to two design criteria: (i) the large cavity eigenmode (beam diameter 8.4 mm × 6.2 mm) on the 1”-diameter mirrors (close to the inner stability edge) prevents damages and achieves a trade-off between losses at the aperture in the output coupler and output coupling efficiency (Fig., 4 shows an estimation of the output coupling efficiency calculated by 3+1 D simulations); (ii) the focus size is maximised under the constraint of an on-axis peak intensity in the range 1–2 × 10^14^ W/cm^2^ (focus radius w_x_ × w_y_ = 25 µm × 32 µm). The gas target was provided by a fused-silica 200-µm end-fire nozzle. Highest XUV flux around 40 eV was achieved with a backing pressure of 4.5 bar of argon gas at the nozzle, which increased the background pressure in the cavity vacuum chamber to 1 × 10^−2^ mbar (a 1600 l/s turbo-molecular pump with a 110-m^3^/h pre-pump were used for evacuation). Reabsorption in the residual gas along the XUV beam path (1.5 m) amounted to 26% (calculated using transmission data from^60^ and our measured XUV spectrum). In order to operate the cavity at its preferred offset frequency of 5.5 MHz^61^, we locked the CEO frequency of the MOPA system to 5.5 MHz using a feed forward scheme^57^. In the presence of the gas target, an intra-cavity power of 2.8 kW at 38-fs pulse duration was achieved over several minutes without any measures of realignment, and over several hours with only minor occasional realignment of the cavity. To prevent mirror contamination, the two cavity mirrors following the HHG target, the NIR/XUV beam splitter and the following gold mirror are constantly flushed with ozone. The cavity lock was implemented according to the Pound-Drever-Hall scheme with two feed-back loops: one slow loop which acts on the cavity length and a fast one adjusting the repetition frequency of the titanium-sapphire oscillator by varying the length of its cavity. To estimate the XUV conversion efficiency in the gas target, we corrected for the output coupling efficiency and for reabsorption in the background gas, resulting in a conversion efficiency of 5 × 10^−8^ at 40 eV. State-of-the art single-pass systems using argon as the generation medium and 1-µm driving pulses^62,63^ reach conversion efficiencies between 5x10^−8^ and 2x10^−7^ at comparable photon energies. The output coupling efficiency (Fig. 4) was estimated with the 3+1 D HHG model described in ref. ^64^ by varying the target gas density and selecting the value with optimum flux around 40 eV, and assuming a gas target position of one Rayleigh range before the focus. This target position was experimentally determined by scanning the nozzle position and observing the clamping of the intracavity power due to plasma-induced blueshift.

### Time-of-flight photoelectron detection

Three different principles are common in electron spectroscopy: retardation of the electrons by a potential barrier (retarding field analyser), dispersion of electrons in a magnetic (or in some cases an electric) field (e.g., hemispherical analysers) and time-of-flight (ToF) analysis. The first two options necessitate a scan over the energy range that is to be analysed, whereas ToF detection allows for the simultaneous detection of electrons of all accepted kinetic energies. More importantly, when combined with a spatially resolving detector, only ToF spectrometers enable the detection of 3D data at once: electron kinetic energy and two dimensions either in space (PEEM) or angle (ARPES). ToF spectrometers are ideal in combination with HHG sources due to the pulsed nature of the latter. The maximum admissible temporal dispersion of electrons is limited by the time between two subsequent laser pulses, which is 54 ns at our repetition rate. Secondary electrons with times of flight far above this value (for any reasonable drift distance) are filtered out by an additional retarding grid as a high-pass filter for electrons. For our drift distance of 880.5 mm (Themis 1000, SPECS GmbH) this typically results in a detectable window of 33–50 eV electron kinetic energy with a resolution of around 30 meV (determined from the measured temporal resolution of our 2D-delayline-detector). This energy range can be increased by accelerating electrons before or within the spectrometer at the expense of energy resolution. Without applying any additional field, our spectrometer collects a solid angle of 0.0016 sr in its so-called drift mode (DM). In its low angular dispersion mode (LAD), a complex multi-element electronic lens system within the spectrometer is used to image a larger solid angle of 0.047 sr on the detector. Since the electron lens system inevitably suffers from chromatic aberration, only a limited energy window can be correctly imaged, and a loss of energy resolution cannot be prevented. Although the earth magnetic field is compensated for down to a residual field of around 2 µT using Helmholtz-coils, this also can have a negative impact on the resolution. We experimentally determined the energy resolution in LAD mode for our settings to be ~1 eV (cf. Fig. 4b). Our instrument resolution can be calculated as the convolution of the detector resolution (30 meV in DM) and the width of the harmonics (between 0.13 eV and 0.35 eV) and amounts to values between 0.13 and 0.36 eV for the individual harmonics. Consequently, space-charge-induced distortions of the photoelectron spectra on the order of a few tens of meV cannot be resolved. In the LAD mode the number of photoelectron counts per second in the observed energy window reached the maximum admissible count rate of 1 MHz on our DLD-detector in its 3D resolved mode. In general, for PES applications a pulse repetition rate high enough so that space charge does not impair the application is desirable. However, an upper bound is set by cumulative effects in the HHG process that limit the XUV yield^65,66^. Furthermore, for ToF-PES there is a trade-off between repetition rate, energy resolution and observable energy window, caused by the ToF dispersion of the electrons. Here, we demonstrate that a repetition rate in the low tens of MHz dramatically increases the PE statistics while also granting a temporal detection duty cycle of nearly 100% and an energy bandwidth and resolution appropriate for solid-state PES while catching all electrons that are relevant for the measurement.

### XUV-IR-interferometer delay stability

To obtain an estimation of the maximum achievable timing precision with our setup, we evaluated the delay stability of the piezo stage (P-621.1CD, Physik Instrumente GmbH) in our interferometer. The position is measured with sub-nanometer resolution using a built-in contact-free capacitive sensor. At a fixed position, in closed loop operation, we found that in 1491 measurements it deviates from the nominal value with a standard deviation of 1.5 nm which is close to the repeatability specified by the manufacturer (±1 nm) and corresponds to a timing jitter of 9.8 as. With state-of-the-art interferometric delay tracking techniques, the delay can in principle be determined with sub-as precision^67^.

## Data Availability

The data that support the findings of this study are available from the corresponding author upon reasonable request.
